# Potential contribution of SIM2 and ETS2 functional polymorphisms in Down syndrome associated malignancies

**DOI:** 10.1186/1471-2350-14-12

**Published:** 2013-01-23

**Authors:** Arpita Chatterjee, Samikshan Dutta, Sanjit Mukherjee, Nupur Mukherjee, Avirup Dutta, Ashis Mukherjee, Swagata Sinha, Chinmay Kumar Panda, Keya Chaudhuri, Ananda L Roy, Kanchan Mukhopadhyay

**Affiliations:** 1 Manovikas Biomedical Research and Diagnostic Centre, MRIH, 482, Madudah, Plot I-24, Sec.-J, E.M. Bypass, Kolkata, 700107, India; 2 Indian Institute of Chemical Biology, Kolkata, India; 3 Chittaranjan National Cancer Institute, Kolkata, India; 4 Netaji Subhash Chandra Bose Cancer Research Institute, Kolkata, India; 5 Tufts University School of Medicine, Boston, MA, USA; 6 Present address: Department of Biochemistry and Molecular Biology, University of Nebraska Medical Center, Nebraska, USA

**Keywords:** SIM2, ETS2, Down syndrome, Breast cancer, Oral cancer, Acute lymphoblastic leukemia

## Abstract

**Background:**

Proper expression and functioning of transcription factors (TFs) are essential for regulation of different traits and thus could be crucial for the development of complex diseases. Subjects with Down syndrome (DS) have a higher incidence of acute lymphoblastic leukemia (ALL) while solid tumors, like breast cancer (BC) and oral cancer (OC), show rare incidences. Triplication of the human chromosome 21 in DS is associated with altered genetic dosage of different TFs. V-ets erythroblastosis virus E26 oncogene homolog 2 (ETS2) and Single Minded 2 (SIM2) are two such TFs that regulate several downstream genes involved in developmental and neurological pathways. Here we studied functional genetic polymorphisms (fSNP) in ETS2 and SIM2 encoding genes in a group of patients and control subjects to better understand association of these variants with DS phenotypes.

**Methods:**

We employed an *in silico* approach to identify potential target pathways of ETS2 and SIM2. fSNPs in genes encoding for these two TFs were identified using available databases. Selected sites were genotyped in individuals with DS, their parents, ALL, BC, OC as well as ethnically matched control individuals. We further analyzed these data by population-based statistical methods.

**Results:**

Allelic/genotypic association analysis showed significant (P < 0.03) differences of rs2070530, rs1051476, rs11254, rs711 for DS subjects compared to control. rs711 also exhibited significantly different genotypic distribution pattern in parents of DS probands (P < 0.02) and BC patients (P < 0.02). Interaction analysis revealed independent main effect of rs711 in all the groups, while rs11254 exhibited independent main effect in DS subjects only. High entropy values were noticed for rs461155 in the solid tumor groups. Significant interactive effects of rs2070531 with rs1051475, rs1051476, rs11254 were observed in all the groups except DS.

**Conclusions:**

We infer from the present investigation that the difference in frequencies of fSNPs and their independent as well as interactive effects may be the cause for altered expression of SIM2 and ETS2 in DS and malignant groups, which affects different downstream biological pathways. Thus, altered expression of SIM2 and ETS2 could be one of the reasons for variable occurrence of different malignant conditions in DS.

## Background

Transcription factors (TFs) regulate pathways related to diseases either through their direct action on the target genes or by controlling downstream pathways. Hence they are important candidates for investigating etiology of complex diseases. There are several TF encoding genes in the human 21^st^ chromosome (HSA21) and deregulated expression of any of these could influence downstream pathways. Due to trisomy of the HSA21 in Down syndrome (DS) (MIM# 190685), genetic overdosage of a number of TF encoding genes is a distinct possibility. DS patients are prone to acute leukemia, including acute lymphoblastic leukemia (ALL), while solid tumors especially breast cancer (BC) is rare [1]. We hypothesized that DS related abnormalities like intellectual disability, immunological imbalance, hormonal alteration, and predisposition to childhood acute leukemia could be due to improper expression and functioning of TFs located in the HSA21. Because disease association studies have revealed higher differential expression ratio in different tissues for the TF genes encoding Single minded 2 (SIM2) and V-ets erythroblastosis virus E26 oncogene homolog 2 (ETS2) within HSA21 [2], here we explored the role of these two TFs in DS phenotype and related malignancies.

SIM2 is important for normal neuronal development. SIM2 can heterodimerize with aryl hydrocarbon receptor nuclear translocator (ARNT) and translocate to the nucleus to transcriptionally regulate gene expression [3]. Expression of SIM2 mRNA has been detected in fetal brain regions associated with DS pathology [4]. SIM2 also plays an important role in carcinogenesis. After entry into a cell, carcinogenic compounds bind to the cytoplasmic Aryl hydrocarbon receptor (AhR) and are carried to the nucleus. Ligand-bound AhR together with ARNT [5] bind to the Xenobiotic Response Element present in the promoter region of certain genes encoding for oxidative enzymes [6-8]; transcriptional activation of these enzymes accelerates carcinogen metabolism [9]. SIM2 inhibits AhR/ARNT dimerization, thereby inhibiting carcinogen metabolism and promoting carcinogenesis [5,10]. In addition, SIM2 is the second most consistently over expressed gene in prostate cancer [11] and over expression of the short isoform of SIM2 (SIM2s) is reported in malignant colon, pancreas, and prostate tissues as compared to the corresponding normal tissues [9-11]. SIM2 has further been proposed to have a breast tumor suppressive activity [12] and a genome-wide linkage scan identified three putative breast cancer susceptibility loci, one of which (21q22) harbors SIM2 [13]. Therefore, SIM2 functions as a tumour selective marker and drug target in several types of malignancies [10].

Besides SIM2, ETS2 over expression induces craniofacial defects as well as skeletal anomalies in transgenic mice resembling DS [14]. Increased rate of neuronal apoptosis [15] and amyloid precursor protein (APP) gene transactivation are also observed upon ETS2 over expression [16], which might play an important role in the early onset of Alzheimer’s disease and neuronal abnormalities in DS [16].

ETS2 can act both as a transcriptional activator as well as a repressor during cellular proliferation, differentiation and tumorigenesis [17-26]. For instance, cell cycle regulator genes like bcl-xL, c-myc, cyclin D1 and p53 are activated by ETS2 [27,28], while BRCA1 expression is repressed in breast cancer tissue [29]. Interestingly, certain genetic translocations in ETS2 were observed in DS patients suffering from leukemia [30]. An interaction of *ETS2* and *ERG* with *GATA1* mutations were reported in DS subjects with acute megakaryoblastic leukemia and activation of the JAK/STAT pathway, a frequent attribute of megakaryocytic malignancies, was identified as common phenomena for this malignant transformation [31].

Given the functional importance of SIM2 and ETS2, we sought out to investigate alterations in their expression in disease etiology. Functional single nucleotide polymorphisms (fSNP) in candidate genes are important indicators for their association with disease phenotypes. In the present study, fSNPs of SIM2 and ETS2 were analyzed for their potential role in Indian individuals suffering from DS, ALL and solid tumors that includes BC and oral cancer (OC).

## Methods

### In Silico analysis to predict pathways regulated by SIM2 and ETS2

We undertook computational methods to determine the probable pathways regulated by SIM2 and ETS2. The promoter sequences (from-5000 bp to +1000 bp) were retrieved from the Eukaryotic promoter database (EPD) (http://www.epd.isb-sib.ch/) and Transcriptional Regulatory Element Database (TRED) (http://rulai.cshl.edu/TRED). Presence of SIM2 and/or ETS2 binding sites in the promoter sequences were identified by a Perl based program “Consensus-Finder”.

**Figure 1:**
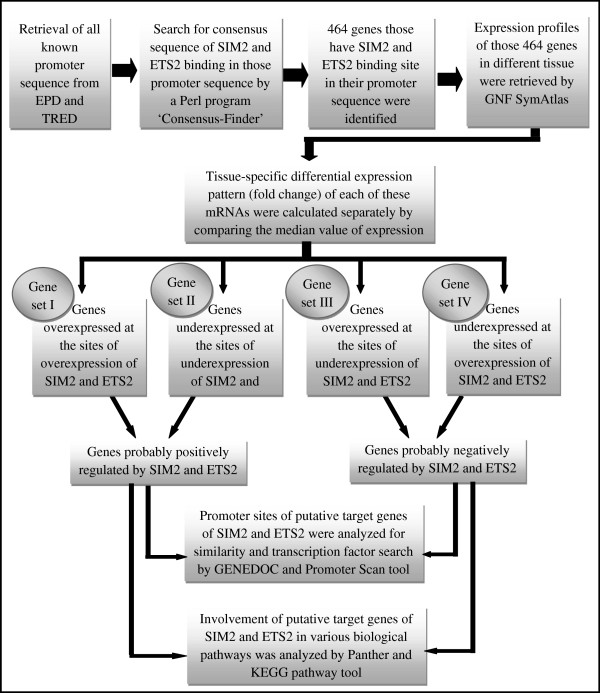
**Schematic presentation of the *in silico *methods used for identification of putative target pathways of SIM2 and ETS2.**

We retrieved expression profile of the genes harboring binding sites for SIM2 and ETS2 in different tissues by using GNF SymAtlas database (http://symatlas.gnf.org/SymAtlas/). Tissue-specific differential expression pattern (fold change) were calculated separately by comparing the median value of expression; value greater than the median was considered as over expression and value less than median was considered as under expression. We then determined co-expression of putative target genes at the site of over expression/under expression of SIM2 and ETS2 and promoter sites of these genes were analyzed by GENEDOC and Promoter Scan tools respectively (http://www-bimas.cit.nih.gov/molbio/proscan/). Functions of these putative target genes along with SIM2 and ETS2 in various biological pathways was analyzed by Panther (http://www.pantherdb.org/pathway/) and KEGG pathway (http://www.genome.ad.jp/kegg/pathway.html). The entire process is presented schematically in Figure 1.

### *In silico* identification of functional variants

We used different web based tools namely SIFT (http://sift.jcvi.org), PolyPhen (http://coot.embl.de/PolyPhen/), SNPs3D (http://www.snps3d.org/), Pupasuite 2 (http://pupasuite.bioinfo.cipf.es), GlobPlot, FastSNP (http://fastsnp.ibms.sinica.edu.tw), SNP@Promoter (http://variome.kobic.re.kr/SNPatPromoter), and dbSMR (http://miracle.igib.res.in/dbSMR) to identify fSNPs in the genomic regions of SIM2 and ETS2 as reported earlier [32]. fSNPs selected for genotyping in the present study are listed in Table 1. 

**Table 1:** Details on SIM2 and ETS2 functional SNPs explored in this study

**Gene**	**SNP ID**	**Position and type**^**a**^	**Alleles (A1/A2)**^**b**^	**Probable function**^**c**^	**MAF (A2) in other populations (CEU, HCB, JPT and YRI)**^**d**^
*SIM2*	rs2269188	Intronic, regulatory	**G**/C	AhR binding site	0.305, 0.567, 0.534, 0.092
	rs2070650	Intronic	**C/**A	C-Myc binding site	0.358,0.578, 0.456, 0.500
	rs79727992	Intronic	**G**/A	NK	0.014 (CEU)
	rs16994404	Syn, Cod	**C**/T	SR protein mediated splicing regulation	100% C (all populations)
	rs77335240	Intronic	**A**/C	NK	No population data
	rs78455239	Intronic	**C**/A	NK	No population data
	rs79022672	Intronic	**G**/A	NK	0.020 (YRI)
*ETS2*	rs34373350	NS, cod	**C**/T	Damaging	0.006 (CEU), 0.025 (YRI)
	rs11700777	Syn, Cod	**A**/G	SR protein mediated splicing regulation	No population data
	rs114481523	NS, cod	**A**/G	NK	0.005 (YRI)
	rs73450556	Intronic	**G**/T	AML-1a banding site	0.500 (YRI)
	rs77688599	Intronic	G/**A**	NK	0.056 (CEU)
	rs60277131	Intronic	**A**/G	NK	0.180 (YRI)
	rs78391361	NS, cod	**C**/T	NK	0.042 (CEU)
	rs1803557	NS, cod	**A**/T	Damaging	No population data
	rs34472454	FS, Cod	-/C	NK	No population data
	rs113798497	Syn, Cod	A/**G**	NK	No population data
	rs374575	Intronic	**C**/T	Transcriptional regulation	0.290, 0.140, 0.041, 0.005
	rs2070529		**C**/T	Cf1, AML-1a binding site	0.168, 0.523, 0.610, 0.270
	rs2070530		**G**/C	V-Myb binding site	0.168, 0.523, 0.610, 0.290
	rs2070531		**C**/T	NIT2 binding site change	0.398, 0.221, 0.227, 0.288
	rs434421		**C**/T	Nkx-2, USF binding	No population data
	rs8128227		**C**/A	NK	0.014 (CEU)
	rs6517481		**A**/G	Ttk 69 binding site	0.361, 0.318 (HCB + JPT), 0.300
	rs79863249		**C**/G	NK	0.011 (HCB + JPT)
	rs60538921		**A**/G	Lyf-1 banding site	No population data
	rs7276961		**A**/G	HSF binding site change	0.347, 0.466 (HCB + JPT), 0.300
	rs1051475	3′UTR	T/**C**	Transcriptional regulation	0.347, 0.227 (HCB + JPT), 0.200
	rs1051476		**C**/G	SR protein mediated Splicing regulation	0.420, 0.239, 0.209, 0.246
	rs72094783		-/GA	NK	No population data
	rs116542090		**G/**A	NK	0.008 (YRI)
	rs74551083		**A**/G	NK	No population data
	rs11540409		**A**/G	NK	No population data
	rs11254		**C**/T	Alteration of miRNA target site	0.432, 0.233, 0.200, 0.250
	rs711		**G/A**	Affects SR protein mediated ESE activity	0.425, 0.200 (HCB + JPT), 0.441

^**a**^*NS* = Nonsynonymous, *Syn* = Synonymous, *Cod* = Coding, *FS* = Frame shift.

^**b**^*A1* = Major allele, *A2* = Minor allele, allele mentioned in **bold** letter is the **ancestral allele**.

^c^*NK* = Not known.

^**d**^*CEU*: Caucasians from Utah with ancestry from western and northern Europe; *YRI*: Yoruba from Ibadan, Nigeria; *HCB*: Han Chinese from Beijing, China and JPT: Japanese from Tokyo, Japan.

### Subjects

Five ethnically matched groups of individuals were recruited for analysis of fSNPs. Healthy volunteers, without any clinical history of intellectual disability or malignant disorder, were recruited as controls (N = 149). Nuclear families having child with DS (N = 132) were recruited from the outpatient department of Manovikas Kendra, Kolkata and trisomic status of the probands was confirmed by karyotyping. ALL patients (N = 38) were recruited from the Netaji Subhash Chandra Bose Cancer Research Institute, Kolkata. Genomic DNA from post-operative normal tissue, adjacent to malignant BC (N = 49) and OC (N = 54) were collected from Chittaranjan National Cancer Research Institute and Indian Institute of Chemical Biology, Kolkata respectively. All samples were acquired after obtaining informed written consent for participation. Institutional Human Ethical Committee approved the study protocol.

### Sample collection, DNA isolation and genotyping

Peripheral blood (~5 ml) collected from control individuals, DS probands, their parents and ALL patients was used for extraction of genomic DNA [33]. Target sequences were amplified and PCR amplicons were subjected to genotyping (Table 2). 

**Table 2:** Genotyping procedure for the studied SNPs

**Gene**	**SNP ID**	**Primer sequence (5′-3′)**	**Genotyping procedure**
*SIM2*	rs2269188	F: CTCACCACGAGCTACCTGAA	RFLP analysis of PCR product using *Bsu36I*
		R: GACCAGGAGAGGGTTTGGTC	
	rs2070650	F: CAGTGCCATGGCCTTTTTAGA	DNA sequence analysis in ABI prism 3130 Genetic Analyzer using Big Dye sequencing kit v3.1 followed by analysis using Sequencing Analysis software v 5.2. Electropherograms obtained were further analyzed by Mutation Surveyor Demo V3.24 software to check for new mutation
	rs79727992	R: CCAATACACACACAGCACCC	
	rs16994404		
	rs77335240		
	rs78455239		
	rs79022672		
*ETS2*	rs34373350	F: GGGGGTTTCCTTCCAGACT	
	rs11700777	R: CTGATTGGGAAAGTCACGTGGG	
	rs114481523		
	rs73450556		
	rs77688599		
	rs60277131		
	rs78391361		
	rs1803557		
	rs34472454		
	rs113798497		
	rs374575	F: GTCTGATCAAGAGGCCCAAG	
	rs2070529	R: CAGAACCACTGGGGAATGAG	
	rs2070530		
	rs2070531		
	rs434421		
	rs8128227		
	rs6517481		
	rs79863249		
	rs60538921		
	rs7276961		
	rs1051475	F: TGTGTTTCTCCGACAGCTCA	
	rs1051476	R: TTTCATCAAGACCCCTACCG	
	rs72094783		
	rs116542090		
	rs74551083		
	rs11540409		
	rs11254	F: CCATTCATTCGGAGAAAACG	RFLP analysis of PCR product using *TaqI*
		R: AAGGCCACGCAGCTAGTAAA	
	rs711	F: GCAACGGCACAGCTAATTCT	RFLP analysis of PCR product using *MspI*
		R: AAATACAACTGTTAAGGGATTCTGA	

### Statistical analyses

Difference in allelic and genotypic frequency of the studied fSNPs in different study groups as compared to control was calculated by simple r x c contingency table (http://www.physics.csbsju.edu/stats/contingency_NROW_NCOLUMN_form.html). Minor allele frequency (MAF) of eleven fSNPs of Indian control individuals was also compared with four major populations studied in the HapMap project [Caucasians from Utah with ancestry from western and northern Europe (CEU), Han Chinese from Beijing, China (HCB) and Japanese from Tokyo, Japan (JPT) and Yoruba from Ibadan, Nigeria (YRI)]. Allelic odds ratios were calculated by Odds ratio calculator (http://www.hutchon.net/ConfidORnulhypo.htm). All P values obtained by allelic and genotypic association test were corrected for multiple testing by PLINK [34] and R program http://www.r-project.org/. Linkage Disequilibrium (LD) between the SNPs was measured by Haploview 4.1 using default settings. Haplotype frequency of fSNPs was inspected by Unphased program (Version 2.404) [35]. Interaction among the genotypes of SIM2 and ETS2 was analyzed by multifactor dimensionality reduction (MDR) software (version 2.0 beta 8.1) [36] and values were expressed as information gain (IG). Power of all chi square tests was calculated by Piface program [37]. Genotype data of four fSNPs (rs461155, rs1051425 in ETS2 and rs2073601, rs2073416 in SIM2) were also included for LD, haplotype and SNP-SNP interaction analysis. For convenience, triplicate homozygous genotypes were considered as diploid homozygous genotypes in DS probands while the triplicate heterozygous genotypes were considered as the diploid heterozygous genotype for all the calculations to compare with respective reference diploid groups [32,38].

## Results

### *In Silico* analysis to predict pathways regulated by SIM2 and ETS2

Computational expression analysis by GNFSymAtlas showed that all the splice variants of SIM2 and ETS2 were over expressed in 13 tissues and under expressed in 12 tissues (Additional file 1: Table S1). Both SIM2 and ETS2 binding sites were identified in 464 genes by the ‘Consensus-Finder’ program from the eukaryotic promoter database. These putative target genes of SIM2 and ETS2 were sorted into four groups (Additional file 1: Table S2). Gene set I contains 71 genes, which showed over expression in all the tissues where SIM2 and ETS2 were also over expressed. Gene set II comprised of 9 genes, which showed down regulation in all the tissues where SIM2 and ETS2 were also down regulated. The 3^rd^ and 4^th^ set of genes exhibited reverse pattern of expression as compared to SIM2 and ETS2. In addition, SP1 and AP2 were identified as common TFs for both SIM2 and ETS2 target genes.

Target pathway identification by Panther and KEGG (Table 3) indicated genes involved in several pathways, including ones related to the development of ectoderm and nervous system (KLK8, LCK, MAG), sensory perception (PDE6D), ionic transport (ATP1A1, ATP1A4, FXYD5, SLC25A2), signal transduction and cell-cell signaling (C1QL1, C4BPA, C4BPB, CEACAM1, FXYD5, LCK, GNB2L1), cell adhesion (MAGEA3, MAGEE1, MAG, LDLR, EMCN, CEACAM1, C4BPB, C4BPA, C1QL1) and induction of apoptosis (LCK, MAGEA3, MAGEE1, PRSS8, SCARB1, TRRAP). Genes governing pathways connected to immunological regulation included ABCB8, ABP1, KLK8, LCK, MAG, PRSS8, RELA, S100A9, SP1, THBS1, TRRAP, and HLA-DOA. Interestingly, genes like KLK8, LCK, RELA, S100A8, S100A9, TRRAP, and GATA3 are known to have roles in malignant development.

**Table 3:** Target genes of SIM2/ETS2 and their probable function identified by in silico analysis

**Gene IDs**	**Location**	**Function (Obtained using Panther Pathway tool)**
A4GALT	22q13.2	Carbohydrate metabolic process, lipid metabolic process, protein metabolic process
ABCB8	7q36.1	Immune system process, extracellular transport, carbohydrate metabolic process, response to toxin
ABP1	7q36.1	Oxidoreductase activity, immune system process, cellular amino acid and derivative metabolic process
ATP1A1	1p13.1	Hydrolase activity, cation transmembrane transporter activity, ion channel activity, cation transport, lipid transport, lipid metabolic process, cellular calcium ion homeostasis
ATP1A4	1q23.2	Cation transport, lipid transport, lipid metabolic process, homeostasis
C1QL1	17q21.31	Complement activation, carbohydrate transport, signal transduction, cell-cell signaling, cell adhesion, carbohydrate metabolic process, lipid metabolic process, cellular component morphogenesis, mesoderm development, skeletal system development, response to stimulus
C4BPA	1q32.2	Complement activation, signal transduction, cell-cell adhesion, protein metabolic process, blood coagulation
C4BPB	1q32.2	Complement activation, signal transduction, cell-cell adhesion, protein metabolic process, blood coagulation
CEACAM1	19q13.2	Signal transduction, cell-cell adhesion
CYB561	17q23.3	Oxidoreductase activity, respiratory electron transport chain
EMCN	4q24	Cell adhesion
EXOSC2	9q34.12	Nucleobase, nucleoside, nucleotide and nucleic acid metabolic process
FVT1	18q21.3	Oxidoreductase activity, metabolic process
FXYD5	19q13.12	Ion channel activity, protein binding ion transport, signal transduction
GATA3	10p14	Hydrolase activity, acting on ester bonds, DNA binding, transcription factor activity nucleobase, nucleoside, nucleotide and nucleic acid metabolic process, endoderm development, heart development, hemopoiesis
GNB2L1	5q35.3	Intracellular protein transport, signal transduction
GTF3C5	9q34.2	DNA binding, nucleobase, nucleoside, nucleotide and nucleic acid metabolic process
H1F0	22q13.1	Nucleobase, nucleoside, nucleotide and nucleic acid metabolic process, organelle organization, establishment or maintenance of chromatin architecture
HLA-DOA	6p21.32	Antigen processing and presentation of peptide or polysaccharide antigen via MHC class II, cellular defense response
HPD	12q24.31	Cellular amino acid and derivative metabolic process
HRB2	12q21.2	RNA binding, nucleobase, nucleoside, nucleotide and nucleic acid metabolic process
KDELR2	7p22.1	Intracellular protein transport, exocytosis
KLK8	19q13.41	Spermatogenesis, immune system process, cell cycle, protein metabolic process, cell cycle, ectoderm development, nervous system development, blood coagulation
KRT16	17q21.2	Structural constituent of cytoskeleton, ectoderm development, cellular component morphogenesis
LCK	1p35.1	Female gamete generation, immune system process, carbohydrate transport, apoptosis, cell cycle, cell surface receptor linked signal transduction, intracellular signaling cascade, carbohydrate metabolic process, protein metabolic process, cell motion, signal transduction, cell-cell signaling, cell-cell adhesion, ectoderm development, mesoderm development, embryonic development, angiogenesis, nervous system development, response to stress
LDLR	19p13.2	female gamete generation, cell adhesion
LPPR4	1p21.2	Cell surface receptor linked signal transduction, phosphate metabolic process, lipid metabolic process, signal transduction
MAG	19q13.12	Receptor activity, structural constituent of myelin sheath, receptor binding, B cell mediated immunity, cell surface receptor linked signal transduction, cell-cell adhesion, signal transduction, cell-cell adhesion, ectoderm development, nervous system development, response to stimulus
MAGEA3	Xq28	Gamete generation, induction of apoptosis, cell adhesion
MAGEE1	Xq13.3	Gamete generation, induction of apoptosis, cell adhesion
MASP1	3q27.3	Complement activation, protein metabolic process, response to stimulus
MLX	17q21.2	DNA binding, nucleobase, nucleoside, nucleotide and nucleic acid metabolic process, transcription factor activity
MOGAT1	2q36.1	Acyl-CoA metabolic process, lipid metabolic process
MRPL37	1p32.3	Protein metabolic process
MRPS12	19q13.2	Protein metabolic process
NAT5	20p11.23	Acyltransferase activity, protein metabolic process
NDUFA2	5q31.3	Oxidoreductase activity, oxidative phosphorylation, respiratory electron transport chain
PCSK4	19p13.3	Peptidase activity, cell surface receptor linked signal transduction, cell-matrix adhesion, protein metabolic process, signal transduction
PDE6D	2q37.1	Visual perception, sensory perception, nucleobase, nucleoside, nucleotide and nucleic acid metabolic process
PRSS8	16p11.2	Peptidase activity, spermatogenesis, immune system process, protein metabolic process
RELA	11q13.1	B cell mediated immunity, negative regulation of apoptosis, cell cycle, intracellular signaling cascade, nucleobase, nucleoside, nucleotide and nucleic acid metabolic process, signal transduction, cellular defense response
RER1	1p36.32	Intracellular protein transport
S100A8	1q21.3	Calcium ion binding, receptor binding, calmodulin binding, immune response, macrophage activation, cell cycle, intracellular signaling cascade, cell motion, cell cycle, signal transduction, response to stimulus
S100A9	1q21.3	Immune response, macrophage activation, cell cycle, intracellular signaling cascade, cell motion, cell cycle, signal transduction, response to stimulus
SCARB1	12q24.31	Receptor activity, macrophage activation, lipid transport, apoptosis, signal transduction, cell adhesion, lipid metabolic process, cellular component morphogenesis
SERPINA1	14q32.13	Protein metabolic process
SFRS1	17q22	RNA splicing factor activity, transesterification mechanism, RNA binding, nucleobase, nucleoside, nucleotide and nucleic acid metabolic process
SLC25A21	14q13.3	Cation transport, phosphate transport, lipid transport, nucleobase, nucleoside, nucleotide and nucleic acid transport, phosphate metabolic process, nucleobase, nucleoside, nucleotide and nucleic acid metabolic process, lipid metabolic process
SLC7A9	19q13.11	Amino acid transmembrane transporter activity, transmembrane transporter activity amino acid transport, cellular amino acid and derivative metabolic process
SP1	12q13.13	Immune system process, nucleoside, nucleotide and nucleic acid metabolic process
TGM2	20q11.23	Protein metabolic process
TH	11p15.5	Oxidoreductase activity, signal transduction, cellular amino acid and derivative metabolic process
THBS1	15q14	Receptor binding, enzyme regulator activity, immune system process, blood coagulation
TRFP	6p21.1	Ubiquitin-protein ligase activity
TRRAP	7q22.1	Immune system process, induction of apoptosis, cell cycle, nucleobase, nucleoside, nucleotide and nucleic acid metabolic process, protein metabolic process, cell cycle, signal transduction, organelle organization, establishment or maintenance of chromatin architecture, response to stress

### In silico identification of functional variants

Different *in silico* tools identified functional genetic variants in SIM2 and ETS2. Among them, thirty five (seven in SIM2 and twenty eight in ETS2) SNPs were genotyped in this study. Functional significance of the SNPs is indicated in Table 1.

### Allelic and genotypic frequency distribution

Comparative analysis of MAF in different populations revealed significant difference in many SNPs (rs374575, rs2070529, rs2070530, rs1051476, rs11254 and rs711 in CEU; rs2269188 and rs7276961 in HCB; rs2269188, rs2070529 and rs2070530 in JPT; rs2269188, rs374575, rs2070529 and rs711 in YRI) (Table 4). Among seven SNPs studied in SIM2 (Table 1), only rs2269188 was polymorphic in the studied population. This SNP showed significant difference in allelic (*χ*^2^ =6.333, P = 0.012, Power = 82.3%) and genotypic (*χ*^2^ =6.41, P = 0.041, Power = 74.17%) frequency only in ALL compared to the control (Additional file 1: Table S3). However, the differences were not significant after correction for multiple testing.

**Table 4:** Minor allele frequencies in different populations as compared to the Indian control individuals

**SNP ID**	**IND MAF**	**CEU MAF**	**Chi Sq, p**	**HCB MAF**	**Chi Sq, p**	**JPT MAF**	**Chi Sq, p**	**YRI MAF**	**Chi Sq, p**
rs2269188	0.348	0.305	0.570, 0.450	0.567	**9.74, 0.002**	0.534	**6.57, 0.010**	0.092	**19.7, 0.000**
rs374575	0.066	0.29	**16.4, 0.000**	0.14	2.61, 0.106	0.041	0.866, 0.352	0.005	**4.69, 0.030**
rs2070529	0.407	0.168	**14.0, 0.000**	0.523	2.43, 0.119	0.61	**8.000, 0.005**	0.27	**4.37, 0.037**
rs2070530	0.417	0.168	**15.0, 0.000**	0.523	2.01, 0.157	0.61	**8.000, 0.005**	0.29	3.69, 0.055
rs2070531	0.293	0.398	2.20, 0.138	0.221	1.29, 0.256	0.227	0.936, 0.333	0.288	0.00, 1.000
rs6517481	0.293	0.361	0.814. 0.367	0.318 (HCB + JPT)		0.212, 0.645		0.3	0.240E-01, 0.877
rs7276961	0.293	0.347	0.570, 0.450	0.466 (HCB + JPT)		**6.88, 0.009**		0.3	0.240E-01, 0.877
rs1051475	0.276	0.347	1.14, 0.287	0.227 (HCB + JPT)		0.658, 0.417		0.2	1.75, 0.185
rs1051476	0.276	0.42	**4.31, 0.038**	0.239	0.416, 0.519	0.209	1.32, 0.25	0.246	0.231, 0.631
rs11254	0.28	0.432	**4.91, 0.027**	0.233	0.658, 0.417	0.2	1.75, 0.185	0.25	0.231, 0.631
rs711	0.26	0.425	**6.39, 0.011**	0.2 (HCB + JPT)		1.02, 0.313		0.441	**7.12, 0.008**

*MAF*: Minor Allele Frequency; *CEU*: Caucasian population; *HCB*: Chinese from Beijing, China; *JPT*: Japanese from Tokyo, Japan; *YRI*: Yoruba from Ibadan, Nigeria.

Twenty eight SNPs in ETS2 genomic region were analyzed and ten of them were polymorphic in the studied population. Eight of the ten ETS2 SNPs (rs374575, rs2070529, rs2070530, rs2070531, rs6517481, rs7276961, rs1051475 and rs1051476) did not show any significant difference in allelic frequency in DS probands, their parent and malignant groups (Additional file 1: Table S3). rs11254 showed a marginal allelic association in DS probands (P = 0.04712) which failed to stand Bonferroni (BF) and Benjamini-Hochberg (BH) correction for multiple testing (Table 5). rs711 showed significant increase in the ‘G’ allele frequency in probands with DS (*χ*^2^ =8.51, BF P and BH P =0.03, Power = 43.47%) as compared to controls. Although a significant increase in the ‘G’ allele (*χ*^2^ =6.83, P = 0.00895, Power = 85.03%, OR = 2.6) was noticed in ALL patients, it was found to be marginally significant after correction for multiple testing (BH P = 0.06). On the other hand, a significant increase in the ‘A’ allele (*χ*^2^ =9.91, BF P and BH P =0.01, Power = 88.26%) was observed in BC patients (Table 5).

**Table 5:** SNPs exhibiting significant differences in allelic and genotypic distribution

**Group**		**Allelic association**					**Genotypic association**		
	**SNP ID (A1/A2)**	**C P**	**BFP**	**BHP**	**OR (CI) for A1**	**OR (CI) for A2**	**C P**	**BFP**	**BHP**
**Father of DS proband (N = 91)**	rs711 (G/A)	**0.019**	0.209	0.215	0.622 (0.417-0.928)	1.608 (1.078-2.398)	**0.002**	**0.022**	**0.022**
**Mother of DS proband (N = 118)**	rs711 (G/A)	**0.00198**	**0.02178**	**0.022**	0.56 (0.387-0.810)	1.785 (1.234-2.581)	**0.001**	**0.011**	**0.011**
**DS proband (N = 132)**	rs2070530 (C/G)	0.892	1	0.892	1.024 (0.729-1.438)	0.977 (0.695-1.372)	**0.003**	**0.033**	**0.015**
	rs1051475 (T/C)	0.314	1	0.576	0.829 (0.576-1.194)	1.206 (0.837-1.736)	**0.026**	0.286	0.057
	rs1051476 (C/G)	0.231	1	0.527	0.801 (0.557-1.152)	1.249 (0.868-1.796)	**0.014**	0.154	**0.039**
	rs11254 (C/T)	**0.04712**	0.51832	0.259	1.487 (1.004-2.201)	0.673 (0.454-0.996)	**0.0001**	**0.0011**	**0.001**
	rs711 (G/A)	**0.00352**	**0.03872**	**0.039**	1.858 (1.221-2.828)	0.538 (0.354-0.819)	**0.004**	**0.044**	**0.015**
**ALL (N = 38)**	rs2269188 (G/C)	**0.01185**	0.13035	0.065	2.17 (1.175-4.008)	0.461 (0.250-0.851)	**0.041**	0.451	0.226
	rs711 (G/A)	**0.00895**	0.09845	0.065	2.617 (1.245-5.501)	0.382 (0.182-0.803)	**0.02**	0.22	0.220
**BC (N = 49)**	rs711 (G/A)	**0.00165**	**0.01815**	**0.018**	0.469 (0.291-0.755)	2.133 (1.324-3.437)	**0.002**	**0.022**	**0.022**
**OC (N = 54)**	rs711 (G/A)	**0.04993**	0.54923	0.549	1.758 (0.995-3.105)	0.569 (0.322-1.005)	0.091	1	0.686

*BFP* = Bonferroni P value, *BHP* = Benjamini-Hochberg P value, *CP* = Crude P value, *CI* = 95% Confidence Interval, *OR* = Odds Ratio.

Significant differences in genotypic frequency for rs11254 (*χ*^2^ = 85.4, P = 0.0001, Power = 99.96%), rs2070530 (*χ*^2^ = 11.5, P = 0.003, Power = 44.76%), rs1051475 (*χ*^2^ =7.33, P = 0.026, Power = 30.01%), rs1051476 (*χ*^2^ =8.57, P =0.014, Power =34.5%) and rs711 (*χ*^2^ =11.2, P =0.004, Power =43.74%) were observed in DS probands. The heterozygous genotype frequency of rs11254 was found to be 0.000 in DS probands. rs711 also showed significant difference in genotype distribution in parents of probands with DS (Father: *χ*^2^ = 12.8, P = 0.002, Power = 65.76%; Mother: *χ*^2^ = 14.6, P = 0.001, Power = 59.81%) as well as in ALL (*χ*^2^ = 7.78, P = 0.02, Power = 82.49%) and BC (*χ*^2^ = 12.1, P = 0.002, Power = 89.24%). ‘AA’ genotype was absent in the ALL and OC groups. Except for rs1051475 in probands with DS and rs711 in ALL patients, the remaining SNPs retained the significance level after BF and BH correction (Table 5).

### LD and haplotype analysis

**Figure 2:**
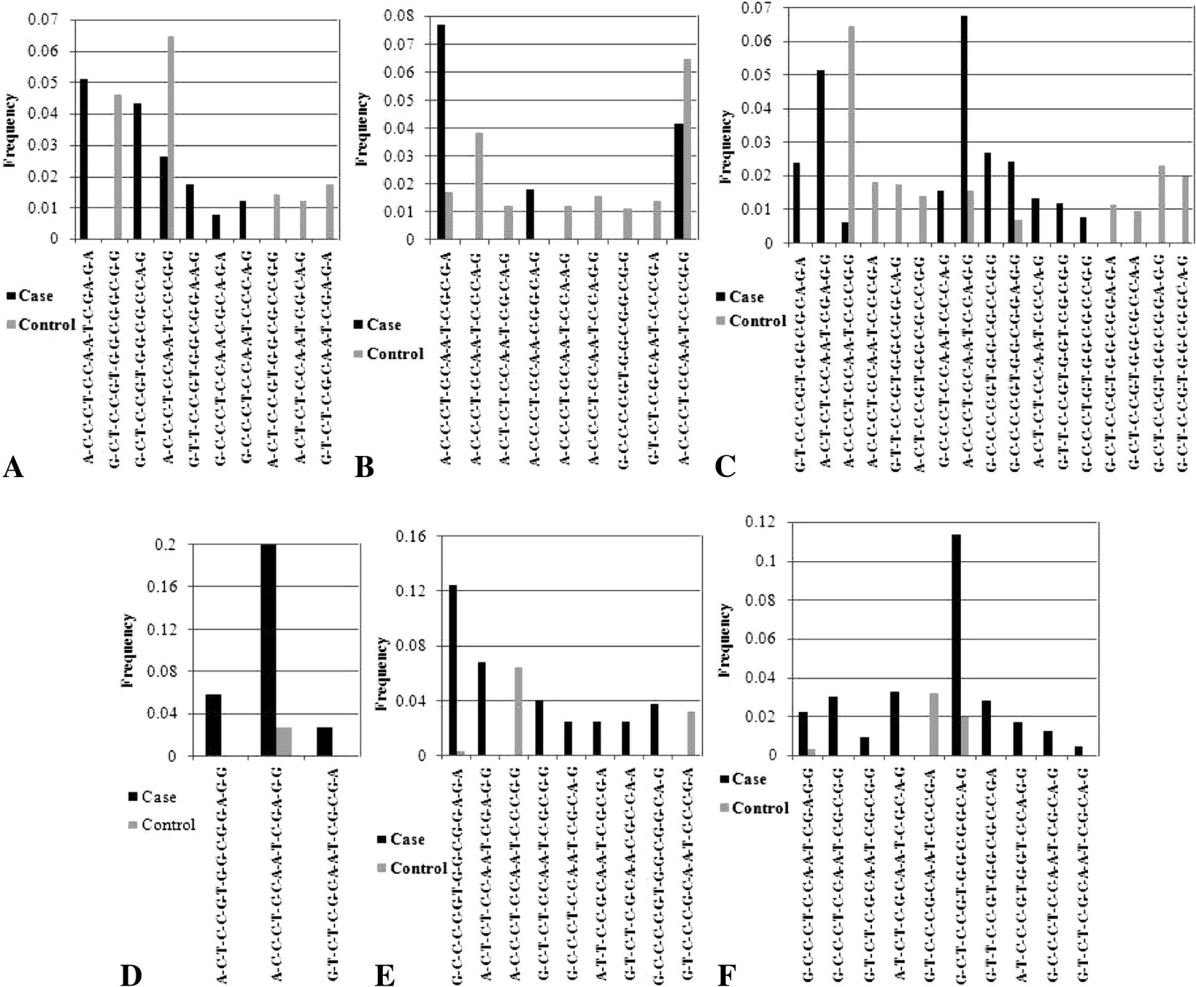
**Haplotypes showing significantly different frequency in Father of probands with DS-DSF (A), Mother of probands with DS-DSM (B), probands with DS- DSP (C), ALL (D), BC (E), OC (F).** Order of the SNPs in the haplotypes is rs461155-rs1051425-rs11254-rs374575-rs2070529-rs2070530-rs2070531-rs6517481-rs7276961-rs1051475-rs1051476-rs2269188-rs2073601-rs2073416-rs711.

SNP pairs that showed higher LD (high D’ or r^2^ value) in at least one combination or different LD patterns in control and case groups during pair wise analysis by Haploview 4.1 were sorted out. In control individuals and parents of probands with DS, all the studied SNPs exhibited strong LD (Table 6). In particular, rs6517481-rs7276961, rs1051475-rs1051476, rs2070529-rs2070530, rs2070531-rs6517481, rs2070531-rs7276961 pairs exhibited strong LD in other studied groups. Some paired combinations showed different LD pattern in different disease groups. For instance, rs11254 showed weak LD with all the sites in DS and BC groups, while rs461155 showed weak LD in OC. Statistically significant differences in frequency of several haplotypes were noticed between test and control groups (Figure 2). Notably the ‘A-C-C-C-T-C-C-A-A-T-C-C-C-G-G’ haplotype showed significant frequency difference in BC, DS proband and their parents when analyzed by Unphased. However, comparison by simple Chi-square test followed by analysis of the power of association by Piface (Additional file 1: Table S4) showed statistically significant difference only for the DS probands and BC groups (p value 0.054 and 0.013 respectively).

**Table 6:** Pair wise LD pattern of studied SNPs

**D′**	**rs6517481**	**rs7276961**	**rs1051475**	**rs1051476**	**rs2070529**	**rs2070530**	**rs2070531**	**rs461155**	**rs11254**
**r**^**2**^									
**Control**									
**rs6517481**	-	1	0.926	0.926	0.909	0.930	0.949	0.735	0.719
**rs7276961**	1	-	0.926	0.926	0.909	0.930	0.949	0.735	0.719
**rs1051475**	0.774	0.774	-	1	0.899	0.923	0.944	0.765	0.722
**rs1051476**	0.774	0.774	1	-	0.899	0.923	0.944	0.765	0.722
**rs2070529**	0.499	0.499	0.441	0.441	-	1	0.955	0.836	0.660
**rs2070530**	0.501	0.501	0.445	0.445	0.958	-	0.977	0.849	0.680
**rs2070531**	0.902	0.902	0.805	0.805	0.551	0.553	-	0.757	0.718
**rs461155**	0.366	0.366	0.362	0.362	0.622	0.615	0.389	-	0.612
**rs11254**	0.491	0.491	0.503	0.503	0.250	0.255	0.490	0.240	-
**Father of DS proband**									
**rs6517481**	-	1	0.856	0.853	0.871	0.954	0.905	0.907	0.845
**rs7276961**	1	-	0.856	0.853	0.871	0.954	0.905	0.907	0.845
**rs1051475**	0.579	0.579	-	1	0.710	0.797	0.888	0.799	0.897
**rs1051476**	0.560	0.560	0.974	-	0.717	0.802	0.886	0.805	0.931
**rs2070529**	0.423	0.423	0.369	0.387	-	0.974	0.956	0.807	0.876
**rs2070530**	0.450	0.450	0.399	0.415	0.841	-	1	0.848	1
**rs2070531**	0.796	0.796	0.606	0.586	0.494	0.479	-	0.903	0.905
**rs461155**	0.417	0.417	0.398	0.414	0.579	0.703	0.400	-	0.861
**rs11254**	0.714	0.714	0.643	0.674	0.442	0.494	0.795	0.376	-
**Mother of DS proband**									
**rs6517481**	-	1	0.828	0.830	0.919	0.918	0.979	0.769	0.916
**rs7276961**	1	-	0.828	0.830	0.919	0.918	0.979	0.769	0.916
**rs1051475**	0.549	0.549	-	1	0.658	0.671	0.849	0.548	0.926
**rs1051476**	0.563	0.563	0.981	-	0.679	0.693	0.849	0.571	0.927
**rs2070529**	0.538	0.568	0.346	0.362	-	1	1	0.745	0.918
**rs2070530**	0.528	0.528	0.346	0.363	0.982	-	1	0.750	0.916
**rs2070531**	0.920	0.920	0.552	0.565	0.612	0.601	-	0.759	0.937
**rs461155**	0.370	0.370	0.228	0.243	0.535	0.563	0.346	-	0.728
**rs11254**	0.823	0.823	0.663	0.677	0.527	0.515	0.861	0.316	-
**DS proband**									
**rs6517481**	-	1	0.865	0.862	0.875	0.975	0.939	0.761	0.255
**rs7276961**	1	-	0.865	0.862	0.875	0.975	0.939	0.761	0.255
**rs1051475**	0.710	0.710	-	1	0.830	0.926	0.903	0.774	0.274
**rs1051476**	0.680	0.680	0.966	-	0.807	0.903	0.885	0.756	0.266
**rs2070529**	0.501	0.501	0.476	0.458	-	0.949	0.825	0.812	0.256
**rs2070530**	0.593	0.593	0.565	0.546	0.859	-	0.911	0.823	0.273
**rs2070531**	0.754	0.754	0.735	0.718	0.523	0.608	-	0.699	0.269
**rs461155**	0.375	0.375	0.410	0.404	0.659	0.645	0.376	-	0.167
**rs11254**	0.039	0.039	0.043	0.039	0.026	0.028	0.037	0.011	-
**ALL**									
**rs6517481**	-	1	1	1	1	1	1	0.761	1
**rs7276961**	1	-	1	1	1	1	1	0.761	1
**rs1051475**	0.939	0.939	-	1	0.926	1	1	0.698	1
**rs1051476**	0.939	0.939	1	-	0.926	1	1	0.698	1
**rs2070529**	0.698	0.698	0.638	0.638	-	1	0.926	0.748	0.858
**rs2070530**	0.625	0.625	0.665	0.665	0.894	-	1	0.731	1
**rs2070531**	0.939	0.939	1	1	0.638	0.665	-	0.698	1
**rs461155**	0.428	0.428	0.383	0.383	0.529	0.451	0.383	-	0.636
**rs11254**	0.883	0.883	0.940	0.940	0.582	0.708	0.940	0.339	-
**BC**									
**rs6517481**	-	1	0.884	0.884	1	1	1	0.5	0.122
**rs7276961**	1	-	0.884	0.884	1	1	1	0.5	0.122
**rs1051475**	0.741	0.741	-	1	1	1	0.884	0.891	0.077
**rs1051476**	0.741	0.741	1	-	1	1	0.884	0.891	0.077
**rs2070529**	0.631	0.631	0.605	0.605	-	1	1	0.495	0.274
**rs2070530**	0.605	0.60	0.582	0.582	1	-	1	0.495	0.269
**rs2070531**	1	1	0.741	0.741	0.631	0.605	-	0.5	0.122
**rs461155**	0.125	0.125	0.350	0.350	0.204	0.204	0.125	-	0.425
**rs11254**	0.013	0.013	0.001	0.001	0.041	0.038	0.013	0.088	-
**OC**									
**rs6517481**	-	1	0.948	0.948	1	1	1	0.334	0.899
**rs7276961**	1	-	0.948	0.948	1	1	1	0.334	0.899
**rs1051475**	0.697	0.697	-	1	1	1	0.950	0.632	0.907
**rs1051476**	0.697	0.697	1	-	1	1	0.950	0.632	0.907
**rs2070529**	0.607	0.607	0.471	0.471	-	1	1	0.391	0.856
**rs2070530**	0.630	0.630	0.488	0.488	0.963	-	1	0.306	0.861
**rs2070531**	0.960	0.960	0.728	0.728	0.582	0.605	-	0.306	0.902
**rs461155**	0.060	0.060	0.168	0.168	0.137	0.049	0.049	-	0.656
**rs11254**	0.656	0.656	0.787	0.787	0.360	0.378	0.687	0.189	-

### Analysis of gene-gene interaction

Gene-gene interaction analysis by MDR 2.0 beta 8.1 indicated that different combinations of SNPs were interacting with each other in different ways within these groups. No highly synergistic interaction was observed in DS probands, while individual effect of different SNPs were found to be high in DS [rs2073601 (1.92%), rs461155 (2.64%), rs1051425 (1.21%), rs2070529 (1.47%), rs2070530 (3.04%), rs2070531 (1.09%), rs6517481 (1.66%), rs7276961 (1.66%), rs1051475 (2.24%), rs1051476 (2.56%), rs11254 (29.78%) and rs711 (4.26%)] (Table 7).

**Table 7:** **Individual and interactive effects of *SIM2 *and *ETS2 *SNPs in families with DS probands (analyzed by MDR 2.0 beta 8.1)**

**SNP ID (individual effect)**	**Interactive effects**														
	**1**	**2**	**3**	**4**	**5**	**6**	**7**	**8**	**9**	**10**	**11**	**12**	**13**	**14**	**15**
**Father**															
rs2073601(**1.77**)	-	−0.06	−1.00	−0.72	−1.49	−1.07	−1.22	−0.82	−1.24	−0.99	−0.99	−0.09	−0.08	−1.12	−1.79
rs2073416 (0.94)		-	−0.65	−0.05	−0.73	−1.12	0.92	−0.37	0.83	−0.46	−0.46	**1.41**	**1.64**	0.72	−0.51
rs2269188 (0.22)			-	1.10	−0.54	−0.38	0.55	1.32	0.50	0.69	0.69	−0.20	−0.07	0.38	0.12
rs461155 (0.70)				-	−0.40	0.39	−0.08	−0.11	0.55	−0.19	−0.19	−0.62	−0.54	−0.40	−0.12
rs1051425 (0.38)					-	−0.58	0.69	−0.18	−0.09	0.55	0.55	0.09	0.19	−0.19	−0.05
rs374575 (0.65)						-	−0.58	−0.38	−0.85	−0.66	−0.66	−0.87	−0.81	−0.57	−0.42
rs2070529 (0.17)							-	−0.14	−0.43	−0.18	−0.18	−0.25	−0.18	−0.67	0.06
rs2070530 (0.44)								-	−0.41	0	0	−0.15	−0.06	**1.16**	−0.21
rs2070531 (0.49)									-	−0.41	−0.41	**3.43**	**3.78**	**2.07**	0.37
rs6517481 (0.34)										-	−0.41	**3.21**	**3.53**	0.37	0.04
rs7276961 (0.34)											-	**3.21**	**3.53**	0.37	0.04
rs1051475 (**2.09**)												-	−2.67	−0.69	0.24
rs1051476 (**2.29**)													-	−0.66	0.46
rs11254 (0.09)														-	−0.11
rs711 (**3.08**)															-
**Mother**															
rs2073601 (**1.38**)	**-**	−1.49	−2.99	−0.16	**−**1.27	−1.38	−0.75	−0.62	−0.14	**−**0.09	−0.09	−0.67	−0.75	−1.31	−1.40
rs2073416 (**1.96**)		-	−4.27	−0.25	−1.45	−1.34	−0.17	−0.17	−0.45	−0.46	−0.46	−0.84	0.03	−0.05	−1.64
rs2269188 (**17.17**)			-	−3.97	−3.97	−4.13	−3.25	−2.90	−3.71	−4.19	−4.19	−5.05	−4.92	−3.47	−4.23
rs461155 (**1.06**)				-	−0.36	0.19	0.02	0.22	0.44	0.49	0.49	−0.46	−0.53	−0.21	−0.84
rs1051425 (**1.11**)					-	−0.95	0.19	−0.25	−1.03	−0.84	−0.84	−0.23	−0.31	−0.84	−0.59
rs374575 (**1.10**)						-	−0.75	−0.41	−0.56	−0.66	−0.66	−0.97	−1.03	−0.69	−0.02
rs2070529 (0.63)							-	−0.54	0.14	0.19	0.19	−0.09	−0.16	**2.27**	0.66
rs2070530 (0.73)								-	0.16	0.3	0.3	0.19	0.07	**2.43**	0.92
rs2070531(0.36)									-	−0.47	−0.47	**2.8**	**2.6**	**5.71**	0.52
rs6517481 (0.30)										-	−0.51	**2.07**	**1.9**	**2.97**	0.73
rs7276961 (0.30)											-	**2.07**	**1.9**	**2.97**	0.73
rs1051475 (**1.47**)												-	−1.71	−0.72	−1.17
rs1051476 (**1.34**)													-	−0.75	−1.04
rs11254 (0.66)														-	−0.67
rs711 (**4.03**)															-
**Probands with DS**															
rs2073601 (**1.92**)	-	−0.37	0.09	−0.34	−0.67	−0.26	−0.81	−0.57	−1.28	−1.14	−1.14	−1.83	−1.74	−3.83	−2.56
rs2073416 (0.26)		-	1.10	−0.22	−0.46	0.12	−0.17	−0.41	−0.39	−1.27	−1.27	−0.49	−0.43	−2.17	−1.72
rs2269188 (0.87)			-	1.35	−0.92	−0.03	0.56	−0.34	0.94	−0.13	−0.13	−0.39	−0.27	−2.78	−2.46
rs461155 (**2.64**)				-	−1.08	0.40	−0.99	−2.07	−0.58	−1.27	−1.27	−1.98	−2.08	−11.9	−3.42
rs1051425 (**1.21**)					-	−0.24	−0.84	−1.36	−0.80	−1.69	−1.69	−0.74	−0.62	−2.60	−0.10
rs374575 (0.03)						-	−0.23	−0.37	0	−0.76	−0.76	−0.48	−0.46	−1.94	−0.47
rs2070529 (**1.47**)							-	−1.61	−0.4	−0.96	−0.96	−0.99	−0.88	−8.74	−1.09
rs2070530 (**3.04**)								-	−0.77	−1.38	−1.38	−1.7	−1.76	−13.5	−1.98
rs2070531 (**1.09**)									-	−1.03	−1.03	−0.8	−0.71	−8.71	−1.31
rs6517481 (**1.66**)										-	−2.73	−1.79	−1.71	−5.3	−2.77
rs7276961 (**1.66**)											-	−1.79	−1.71	−5.3	−2.77
rs1051475 (**2.24**)												-	−2.68	−7.08	−2.15
rs1051476 (**2.56**)													-	−6.21	−2.47
rs11254 (**29.78**)														-	−3.58
rs711(**4.26**)															-

1 = rs2073601, 2 = rs2073416, 3 = rs2269188, 4 = rs461155, 5 = rs1051425, 6 = rs374575, 7 = rs2070529, 8 = rs2070530, 9 = rs2070531, 10 = rs6517481, 11 = rs7276961, 12 = rs1051475, 13 = rs1051476, 14 = rs11254, 15 = rs711 (Entropy values mentioned in bold are in synergistic interaction).

While there was no high individual effect of rs2070529, rs2070530, rs2070531, rs6517481 and rs7276961, high synergistic interaction of these SNPs with rs11254 was noticed in mother of probands with DS (Table 7). In father of probands with DS, rs2070531, rs6517481 and rs7276961 also made a cluster together with rs2073416. High individual effect of rs2073601 (1.77%), rs1051475 (2.09%), rs1051476 (2.29%) and rs711 (3.08%) was observed in father of probands with DS (Table 7), while rs2073601 (1.38%), rs2073416 (1.96%), rs2269188 (17.17%), rs461155 (1.06%), rs1051425 (1.11%), rs374575 (1.10%), rs1051475 (1.47%), rs1051476 (1.34%) and rs711 (4.03%) showed high individual effect in mothers (Table 7).

In the malignant groups, rs2070530, rs2070531, rs6517481, rs7276961, rs1051475, rs1051476 and rs11254 showed synergistic interaction (IG values are mentioned in Table 8). Many of the studied fSNPs also showed significant individual effect in ALL [rs2073601 (3.69%), rs2073416 (2.95%), rs2269188 (2.56%), rs374575 (1.03%) and rs711 (3.55%)], BC [rs2073601 (3.50%), rs2073416 (2.17%), rs461155 (15.15%), rs374575 (4.04%), rs2070529 (3.79%), rs2070530 (3.03%), rs2070531 (4.01%), rs6517481 (3.88%), rs7276961 (3.88%), rs1051475 (1.00%), rs1051476 (1.00%), rs11254 (1.12%) and rs711 (4.56%)] and OC [rs2073601 (2.04%), rs461155 (13.14%), rs374575 (1.89%), rs2070529 (1.16%), rs2070531 (1.47%), rs6517481 (1.33%), rs7276961 (1.33%) and rs711 (2.26%)] (Table 8).

**Table 8:** **Individual and interactive effects of *SIM2 *and *ETS2 *SNPs in different malignant groups (analyzed by MDR 2.0 beta 8.1)**

	**Interactive effects**														
**SNP ID (individual effect)**	**1**	**2**	**3**	**4**	**5**	**6**	**7**	**8**	**9**	**10**	**11**	**12**	**13**	**14**	**15**
**ALL**															
rs2073601 (**3.69**)	-	−4.91	−3.23	−1.16	−0.45	−3.42	−1.34	−1.41	−2.55	−2.33	−2.33	−2.45	−2.45	−2.13	−4.27
rs2073416 (**2.95**)		-	−1.55	−1.00	−1.97	−2.21	−1.99	−2.25	−2.44	−1.98	−1.98	−2.12	−2.12	−1.58	−3.53
rs2269188 (**2.56**)			-	0.74	−0.29	−0.01	0.79	0.16	−0.47	−0.25	−0.25	−0.22	−0.22	−0.31	−2.15
rs461155 (0.07)				-	0.21	−0.53	−0.37	0.30	−0.63	−0.39	−0.39	−0.21	−0.21	−0.29	−0.65
rs1051425 (0.65)					-	−0.31	−0.13	−0.52	−0.06	0.18	0.18	0.07	0.07	−0.26	−0.74
rs374575 (**1.03**)						-	−1.08	−1.18	−1.15	−1.14	−1.14	−0.89	−0.89	−1.06	−0.69
rs2070529 (0.73)							-	−1.04	−0.91	−0.16	−0.16	0.48	0.48	0.91	−1.15
rs2070530 (0.64)								-	−1.10	−0.90	−0.90	0.15	0.15	**1.87**	−1.06
rs2070531 (0.95)									-	−1.13	−1.13	**1.44**	**1.44**	**2.53**	−1.23
rs6517481 (0.58)										-	−1.09	**2.21**	**2.21**	**2.90**	−1.00
rs7276961 (0.58)											-	**2.21**	**2.21**	**2.90**	−1.00
rs1051475 (0.39)												-	−0.57	**2.52**	−0.97
rs1051476 (0.39)													-	**2.52**	−0.97
rs11254 (0.63)														-	−0.97
rs711 (**3.55**)															-
**BC**															
rs2073601 (**3.50**)	**-**	−4.62	−3.32	−3.69	**−**3.32	−3.33	−3.89	−3.12	−3.03	−2.66	−2.66	−1.97	−1.97	−0.76	−3.03
rs2073416 (**2.17**)		-	−2.10	−2.36	−1.86	−1.78	−4.05	−3.14	2.60	2.47	2.47	1.90	1.90	−0.80	−1.56
rs2269188 (0.92)			-	−2.70	**1.66**	0.07	−1.09	−0.14	−2.90	−2.77	−2.77	−0.96	−0.96	−1.45	0.02
rs461155 (**15.15**)				-	−0.77	−3.55	−3.99	−3.22	−4.20	−4.07	−4.07	−1.19	−1.19	−1.31	−7.00
rs1051425 (0.58)					-	−2.71	−1.57	−0.97	−0.97	−1.50	−1.50	0.37	0.37	−0.66	−1.16
rs374575 (**4.04**)						-	−5.88	−5.44	−3.83	−4.28	−4.28	−2.39	−2.39	−2.19	−3.01
rs2070529 (**3.79**)							-	−5.97	−3.87	−5.35	−5.35	−1.63	−1.63	−0.79	−2.14
rs2070530 (**3.03**)								-	−4.90	−5.15	−5.15	−1.54	−1.54	0.33	−1.63
rs2070531 (**4.01**)									-	−4.91	−4.91	**2.19**	**2.19**	**3.81**	−2.37
rs6517481 (**3.88**)										-	−5.09	**0.86**	**0.86**	**3.94**	−1.95
rs7276961 (**3.88**)											-	**0.86**	**0.86**	**3.94**	−1.95
rs1051475 (**1.00**)												-	−1.23	**7.32**	0.19
rs1051476 (**1.00**)													-	**7.32**	0.19
rs11254 (**1.12**)														-	−1.36
rs711 (**4.56**)															-
**OC**															
rs2073601 (**2.04**)	-	−0.91	−1.51	−2.04	−0.62	−1.12	−0.79	−0.79	−1.14	−1.27	−1.27	−1.46	−1.46	−1.38	−2.44
rs2073416 (0.04)		-	0.21	−0.41	−0.25	−1.23	0.24	**1.00**	−0.58	−0.37	−0.37	**1.27**	**1.27**	0.42	−1.05
rs2269188 (0.81)			-	−1.17	0.45	−0.52	−0.49	−0.35	−0.97	−0.94	−0.94	−0.17	−0.17	−0.26	−1.31
rs461155 **(13.14**)				-	−0.70	−1.58	−0.64	−0.38	−1.83	−1.69	−1.69	0.38	0.38	−0.47	−1.26
rs1051425 (0.34)					-	−1.38	−0.77	−0.76	−1.37	−1.07	−1.07	−0.05	−0.05	−0.20	−1.23
rs374575 (**1.89**)						-	−2.33	−2.23	−1.45	−1.50	−1.50	−1.56	−1.56	−0.97	−2.66
rs2070529 (**1.16**)							-	−1.34	−1.78	−1.47	−1.47	−1.05	−1.05	−0.35	−1.52
rs2070530 (0.91)								-	−1.68	−1.31	−1.31	−1.92	−1.92	−0.44	−1.63
rs2070531 (**1.47**)									-	−2.48	−2.48	**1.90**	**1.90**	0.67	−2.13
rs6517481 (**1.33**)										-	−2.40	−1.46	−1.46	0.03	−1.99
rs7276961 (**1.33**)											-	−1.46	−1.46	0.03	−1.99
rs1051475 (0.48)												-	−0.69	**1.33**	−0.73
rs1051476 (0.48)													-	**1.33**	−0.73
rs11254 (0.11)														-	−0.51
rs711 (**2.26**)															-

1 = rs2073601, 2 = rs2073416, 3 = rs2269188, 4 = rs461155, 5 = rs1051425, 6 = rs374575, 7 = rs2070529, 8 = rs2070530, 9 = rs2070531, 10 = rs6517481, 11 = rs7276961, 12 = rs1051475, 13 = rs1051476, 14 = rs11254, 15 = rs711 (Entropy values mentioned in bold are in synergistic interaction).

## Discussion

The present study was aimed at identifying possible involvement of SIM2 and ETS2, two TFs known to have gene overdosage in probands with DS exhibiting trisomy of HSA21. To identify SIM2 and ETS2 targets, we focused on 464 genes containing binding site for both these factors in their regulatory regions (−5000 bp to +1000 bp). Following categorization based on expression pattern by GNF SymAtlas, 91 genes were identified as up- or down regulated by these TFs (Additional file 1: Table S2). Genes like ABP1, HRB2, S100A8, THBS1, CYB561, GATA1, GATA3, SP1 and AP2 indicated potential activation by SIM2 and ETS2 (gene set I and II), while genes such as GCNT2, MASP1, LOC338328, PCSK4, ICAM1, LPPR4, SLC25A21, H1F0 and ATP1A1 indicated potential repression by these TFs (gene set III and IV). Many of the genes with binding sites for SIM2 and ETS2, viz. KLK8, LCK, TRRAP, GATA3, etc. were earlier reported to have role in neurological as well as malignancy related pathways [28,39,40]. Analysis in the present study by Panther also revealed that genes such as KLK8, KRT16, and LCK carrying binding sites for SIM2 and ETS2, are involved in the development and function of the neurological system. Hence over expression of SIM2 and ETS2 might alter expression of the downstream target genes leading to different DS phenotypes.

Previous analysis of DS revealed ambiguous observations on expressions of genes in HSA21 and other autosomes. For instance, a dosage dependent increase in transcription across different tissue/cell types was noticed in DS [41]. Analysis of lymphoblastoid cell lines generated from unrelated individuals revealed over expression of several HSA21 genes even in normal healthy volunteers [42]. In contrast, gene expression profile analysis of hearts of human fetuses with trisomy of HSA21 showed significant downregulation of 278 genes and upregulation of 195 genes as compared to controls [43]. On the other hand, serial analysis of gene expression in lymphocytes from children with DS revealed modest deregulation of autosomal genes [44]. Whole genome microarray in adult DS brains showed upregulation of 27% of genes on HSA21 as compared to 4.4% of genes on other autosomes [45]. Contrary to that, microarray analysis of cultured amniocytes and chorionic villus cells from fetuses with trisomy 13, 18, or 21 revealed lack of over expression of most of the HSA21 genes with only modest changes for genes on all other chromosomes [46]. It is possible that the differences in gene expression in HSA21 and other autosomes are due to the tissue of origin [47].

Differential expression of SIM2 and ETS2 target genes was also reported in different malignancies. For instance, the TRRAP gene, involved in transcriptional regulation and DNA repair, was found to be high in bone metastases from prostate cancer, intermediate in BC, and low in lung and kidney cancers [39]. KLK8 was upregulated in colorectal cancer and ovarian cancer while underexpressed in esophageal and cervical cancer [40,48]. Differential gene expression profiling of approximately 8000 genes in sixty different cancer cell lines revealed difference in gene expression pattern to be correlated with the tissue of origin and the physiological properties (e.g., doubling time, drug metabolism, and interferon response) of cell lines [49]. Difference in expression between specific cancer cell line and their nonmalignant counterparts was also noticed [49]. It is intriguing to note that genes like MAGEA3 and ATP1A1, which indicated potential over expression in our study, are also over expressed in leukemia/lymphoma [48,50-52]. However, THBS1, which also indicated potential upregulation in the present study, was down regulated in leukemia and upregulated in lymphoma [48,53]. Thus, it remains unclear whether differential expression is also taking place for genes identified by our present *in silico* analysis. Further validation, involving expression analysis in various tumor tissues of individuals with DS, will be necessary.

Our next goal was to identify fSNPs in these two TFs. A number of SNPs with deleterious effects were identified in both the genes by our *in silico* approach. Analysis of allelic frequencies showed significant difference in MAF for the Indian control population as compared to other Asian, i.e. Japanese and Chinese, as well as Caucasian populations. Frequency distribution analysis revealed that the rs2269188 ‘G’ allele was significantly high in ALL subjects, which failed to stand test for multiple correction. Haplotypes showing significant difference in ALL, BC and OC groups harbored the rs2269188 ‘G’ allele. MDR analysis revealed high individual effect of this SNP in ALL (2.56%) and in mother of DS probands (17.17%) but not in any other groups. ‘G’ allele is responsible for AhR binding to SIM2. AhR binding with ARNT is an important step for carcinogen metabolism, which is inhibited by SIM2 [6-10]. We speculate that increased frequency of the rs2269188 ‘G’ allele may result in inappropriate metabolism of carcinogenic compounds, thus contributing to the development of leukemia.

rs711 is a site for SR protein mediated splicing regulation and may generate splice variants. In the Korean population, rs711 was reported to be associated with increased risk for acute myeloid leukemia [54]. In the present study, difference in allelic frequency for this site showed a trend to be significant in ALL even after correction for multiple testing, while DS probands, parents of DS probands and BC showed significant differences. MDR analysis supported evidence of individual effect of this SNP in all the studied groups.

rs11254, rs2070530 and rs1051476 showed significant difference in genotype distribution in DS probands (BH P = 0.001, 0.01 and 0.03 respectively). Though there was individual effect of these SNPs (29.78%, 3.04%, and 2.56% respectively), no significant synergistic effect was observed. rs11254 showed a very high individual effect (29.78%) in DS probands which could be due to 100% reduction in heterozygosity. On the other hand in malignant groups, rs11254 showed interactive effect in synergistic mode with other SNPs (rs2070530, rs2070531, rs6517481, rs7276961, rs1051475 and rs1051476). Therefore, this SNP may act differently in DS and other malignant groups.

While comparing differences in haplotype frequencies generated by fifteen SNPs, we analyzed each pair by simple Chi square tests to avoid errors due to multiple comparisons. The ‘A-C-C-C-T-C-C-A-A-T-C-C-C-G-G’ haplotype showed statistically significant higher occurrence in the control group compared to DS probands and BC. Frequency of this haplotype was also higher compared to other haplotypes generated from these 15 SNPs, which may be conferring protection towards the diseases.

MDR analysis exhibited high individual entropy value for rs461155 in both BC and OC groups. Involvement of risk allele of rs461155 in subjects with these two solid tumors has also been reported earlier [32]. Therefore, from the present study we predict that rs461155 may individually play an important role in solid tumor groups (BC and OC). On the other hand, rs2070530, rs2070531, rs6517481, rs7276961, rs1051475, rs1051476 and rs11254 may act together in ALL, BC and OC groups, where rs11254 act as a nodal SNP. *In silico* analysis revealed that, rs11254 has a potency to change miRNA and TF binding sites in the 3'UTR of ETS2. Presence of risk allele and inappropriate interaction of rs11254 probably can hamper proper expression of ETS2. There are various reports on loss of heterozygosity (LOH) of different genes under different malignant conditions like ovarian tumors [55], BC [56], head and neck squamous cell carcinoma [57], pituitary tumors [58], AML [59] etc. We found 100% LOH for rs11254 in DS probands.

Analysis of LD pattern of studied SNPs exhibited that rs6517481, rs7276961, rs1051475, rs1051476, rs2070529, rs2070530, rs2070531, rs461155 and rs11254 are in high LD in the studied population. MDR analysis also provided evidence of interaction between these SNPs in the malignant groups and parents of probands with DS and thus, may suggest combined effect of these fSNPs in the studied groups.

Similar to the present observation, SNP pairs rs2070529-rs2070530 were found to be in high LD in other populations studied in the HapMap; LD data for other SNP pairs were not available. Both haplotype distribution pattern and LD between different SNPs were found to vary in different groups examined in the present investigation, which could be attributed to the difference in allelic frequencies. Whether the observed difference is contributing to the disease etiology requires further analysis.

Our results do not imply that ETS2 and SIM2 are the only TFs in the HSA21 with a role in oncogenesis because several other TFs, located in the HSA21, also have association with malignancies [31,60]. For example, increased expression of BACH1 (transcriptional regulator of megakaryocytic differentiation process) and SON (homologous sequence with MYC family of oncoproteins) were reported in association with myeloid leukemia in DS [61]. RUNX1 and ERG were hypothesized as candidates for leukemia in non-DS patients; however, triplicate dosages of these two genes were incapable to generate transient myeloproliferative leukemia in Ts1Cje mice and thus, these two genes may not be directly responsible for development of leukemia in individuals with DS [62]. Further analysis of these TFs, in association with SIM2 and ETS2, would help us to understand their actual role in DS associated malignancies.

## Conclusions

We summarize that, a) the rs2269188 ‘G’ allele, showing trend for higher occurrence in ALL patients (BH P = 0.06, OR = 2.6), may play a regulatory role in ALL by altering carcinogen metabolism; in mother of probands with DS also, this SNP may contribute some regulatory role as the individual effect of this SNP calculated by MDR analysis was very high (Table 7); that b) rs711 may have very important role in DS and associated malignancies; that c) the fSNP rs11254 may act as a core SNP in the interaction cluster of rs6517481, rs7276961, rs1051475, rs1051476, rs2070529, rs2070530 and rs2070531, thus playing a role in malignant development in BC, OC, ALL; in parents of DS probands, these SNPs also showed strong interaction while in DS, a high individual effect of rs11254 was found; and that d) rs2070530, rs711 and rs11254 (with 100% LOH) showed strong genotypic association with DS. This prominent difference in status of fSNPs of SIM2 and ETS2 may indicate a significantly different pattern of SIM2 and ETS2 regulation in the studied groups, eventually leading to altered expression of their downstream genes associated with distinct disease phenotypes.

## Competing interests

The authors declare that they have no competing interests.

## Authors’ contributions

AC: Concept and designing, genotyping, analysis and manuscript preparation. SD: Karyotyping and confirmation of trisomy 21. SM: Collection and DNA isolation of OC samples. NM: Collection and DNA isolation of BC samples. AD: Programming and running of “Consensus-Finder”. AM: Recruitment of ALL patients. SS: Recruitment of DS patients. CKP: Supervision of NM’s work, consultation during manuscript preparation. KC: Supervision of SM and AD’s work, input in manuscript preparation. ALR: Critical evaluation and editing. KM: Concept and design of the work, supervision of whole process, manuscript preparation and final revision. All authors read and approved the final manuscript.

## Pre-publication history

The pre-publication history for this paper can be accessed here:

http://www.biomedcentral.com/1471-2350/14/12/prepub

## Supplementary Material

Additional file 1 **Table S1.** Sites of overexpression and underexpression of SIM2 and ETS2. **S2**: Possible downstream genes of SIM2 and ETS2 identified by *in silico* analysis. **S3**: Details of allelic and genotypic association test of studied SNPs. **S4**: Comparative analysis of haplotypes in different study group. Click here for file
