# Supplementary material for: Potential contribution of SIM2 and ETS2 functional polymorphisms in Down syndrome associated malignancies
Source: BMC Med Genet. 2013 Jan 23;14:12. doi: 10.1186/1471-2350-14-12 (PMC3563522; doi:10.1186/1471-2350-14-12)
Supplement: Additional file 1 — Table S1. Sites of overexpression and underexpression of SIM2 and ETS2. S2: Possible downstream genes of SIM2 and ETS2 identified by in silico analysis. S3: Details of allelic and genotypic association test of studied SNPs. S4: Comparative analysis of haplotypes in different study group. [file 1471-2350-14-12-S1.doc]

**Table S1. Sites of overexpression and underexpression of SIM2 and ETS2**

| **Serial No** | **Sites of overexpression** | **Sites of underexpression** |
| --- | --- | --- |
| 1 | 721_B_lymphoblasts | Adipocytes |
| 2 | BM-CD105^+^Endothelial cells | Cerebellum |
| 3 | BM-CD33^+^Myeloid cells | Pons |
| 4 | BM-CD34^+^ cells | Testis |
| 5 | Cardiac Myocytes | Testis Germ Cell |
| 6 | Lung | Testis Interstitial |
| 7 | PB-CD14^+^Monocytes | Testis and Seminiferous Tubule |
| 8 | Placenta | Caudate nucleus |
| 9 | Pancreatic Islets | Fetal liver |
| 10 | Prefrontal Cortex | Lymphoblastic Leukemia (molt4) |
| 11 | Prostate | Promyelocytic Leukemia (hl60) |
| 12 | Thyroid | Spinal cord |
| 13 | Uterus Corpus | - |

**Table S2. Possible downstream genes of SIM2 and ETS2 identified by *in silico* analysis**

| **Category** | **Expression pattern** | **Genes selected as targets for SIM2 and ETS2** | **Expression pattern*** |
| --- | --- | --- | --- |
| **I** | **SIM2 ­**  **ETS2­** | A4GALT, ABCB8, ABP1, AK2, AMH, C1QL1, C4BPA, C4BPB, CIRBP, CYB561, DDX49, EMCN, EXOSC2, FLJ10458, FVT1, FXYD5, GNAI2, GNB2L1, GTF3C5, HLA-DOA, HNMT, HPD, HRB2, KDELR2, KLK8, KRT16, LCK, LDLR, MAG, MAGEA3, MAGEC2, MGC11061, MLX, MRAP, MRPS12, NA, NAT5, NDUFA2, NUDC, PDE6D, PDGFB, PLOD1, PPIE, PPP1R15A, PRSS8, PTPRCAP, RELA, RER1, S100A8, S100A9, SCARB1, SERPINA1, SERPINC1, SFRS1, SLC7A9, SNX1, STC2, T1A-2, TAF11, TAS2R14, TBL2, TGM2, TH, THBS1, TM7SF4, TRFP, TRRAP, TXNL2, GATA1, GATA3, SP1, AP2 | **­** |
| **II** | **SIM2¯**  **ETS2¯** | ABP1, NFKBIA, PRF1, HRB2, S100A8, THBS1, CYB561, GATA1, GATA3, SP1,AP2 (in most of the tissues) | **↓** |
| **III** | **SIM2­**  **ETS2 ­** | GCNT2, MASP1, FLJ22490, LOC338328, PCSK4, FLJ31795, CEACAM1, ICAM1, LPPR4, MAGEE1, MOGAT1, SLC25A21, H1F0, MRPL37, ATP1A1, ATP1A4 | **↓** |
| **IV** | **SIM2 ↓**  **ETS2¯** | CPSF3, GCNT2, MASP1, LOC338328, PCSK4, ICAM1, LPPR4, SLC25A21, H1F0, ATP1A1 | **­** |

*Upward arrow denotes over expression while downward arrow denotes under expression of the genes

**Table S3. Details of allelic and genotypic association test of studied SNPs**

| **Group** |  | **Allelic Association** | | | | | **Genotypic Association** | | |
| --- | --- | --- | --- | --- | --- | --- | --- | --- | --- |
| **Father of DS proband (N=91)** | **SNP (A1/A2)** | **C P** | **BF P** | **BH P** | **OR (CI) [for A1]** | **OR (CI) [for A2]** | **C P** | **BF P** | **BH P** |
|  | rs2269188 (G/C) | 0.408 | 1 | 0.665 | 1.182 (0.795-1.756) | 0.846 (0.570-1.257) | 0.693 | 1 | 0.843 |
|  | rs374575 (C/T) | 0.897 | 1 | 0.942 | 1.052 (0.488-2.265) | 0.951 (0.441-2.048) | 0.485 | 1 | 0.843 |
|  | rs2070529 (T/C) | 0.575 | 1 | 0.791 | 1.116 (0.760-1.639) | 0.896 (0.610-1.315) | 0.766 | 1 | 0.843 |
|  | rs2070530 (C/G) | 0.942 | 1 | 0.942 | 1.014 (0.693-1.485) | 0.986 (0.673-1.444) | 0.587 | 1 | 0.843 |
|  | rs2070531 (C/T) | 0.349 | 1 | 0.665 | 1.225 (0.801-1.874) | 0.816 (0.534-1.249) | 0.553 | 1 | 0.843 |
|  | rs6517481 (A/G) | 0.423 | 1 | 0.665 | 1.189 (0.779-1.815) | 0.841 (0.551-1.285) | 0.693 | 1 | 0.843 |
|  | rs7276961 (A/G) | 0.423 | 1 | 0.665 | 1.189 (0.779-1.815) | 0.841 (0.551-1.285) | 0.694 | 1 | 0.843 |
|  | rs1051475 (T/C) | 0.422 | 1 | 0.665 | 0.845 (0.560-1.274) | 1.183 (0.785-1.784) | 0.104 | 1 | 0.381 |
|  | rs1051476 (C/G) | 0.35 | 1 | 0.665 | 0.823 (0.546-1.239) | 1.215 (0.807-1.830) | 0.075 | 0.825 | 0.381 |
|  | rs11254 (C/T) | 0.647 | 1 | 0.791 | 1.103 (0.726-1.675) | 0.907 (0.597-1.378) | 0.867 | 1 | 0.867 |
|  | rs711 (G/A) | **0.019** | 0.209 | 0.215 | 0.622 (0.417-0.928) | 1.608 (1.078-2.398) | **0.002** | **0.022** | **0.022** |
| **Mother of DS proband**  **(N=118)** | rs2269188 (G/C) | 0.055 | 1 | 0.391 | 1.446 (0.992-2.108) | 0.692 (0.474-1.008) | 0.16 | 1 | 0.509 |
|  | rs374575 (C/T) | 0.768 | 1 | 0.984 | 0.903 (0.458-1.779) | 1.107 (0.562-2.181) | 0.776 | 1 | 0.776 |
|  | rs2070529 (T/C) | 0.938 | 1 | 0.984 | 0.986 (0.695-1.400) | 1.014 (0.714-1.439) | 0.438 | 1 | 0.639 |
|  | rs2070530 (C/G) | 0.984 | 1 | 0.984 | 0.996 (0.702-1.414) | 1.004 (0.707-1.424) | 0.268 | 1 | 0.543 |
|  | rs2070531 (C/T) | 0.83 | 1 | 0.984 | 0.96 (0.658-1.400) | 1.042 (0.714-1.520) | 0.528 | 1 | 0.639 |
|  | rs6517481 (A/G) | 0.67 | 1 | 0.984 | 0.92 (0.633-1.342) | 1.085 (0.745-1.581) | 0.581 | 1 | 0.639 |
|  | rs7276961 (A/G) | 0.67 | 1 | 0.984 | 0.92 (0.633-1.342) | 1.085 (0.745-1.581) | 0.581 | 1 | 0.639 |
|  | rs1051475 (T/C) | 0.059 | 1 | 0.275 | 0.7 (0.482-1.015) | 1.43 (0.985-2.075) | 0.146 | 1 | 0.509 |
|  | rs1051476 (C/G) | 0.075 | 1 | 0.275 | 0.713 (0.491-1.035) | 1.104 (0.966-2.036) | 0.185 | 1 | 0.509 |
|  | rs11254 (C/T) | 0.606 | 1 | 0.984 | 0.906 (0.621-1.319) | 1.104 (0.758-1.609) | 0.296 | 1 | 0.543 |
|  | rs711 (G/A) | **0.00198** | **0.02178** | **0.022** | 0.56 (0.387-0.810) | 1.785 (1.234-2.581) | **0.001** | **0.011** | **0.011** |
| **DS proband**  **(N=132)** | rs2269188 (G/C) | 0.114 | 1 | 0.418 | 0.759(0.539-1.069) | 1.318 (0.936-1.856) | 0.186 | 1 | 0.256 |
|  | rs374575 (C/T) | 0.862 | 1 | 0.892 | 0.943(0.484-1.838) | 1.061 (0.544-2.068) | 0.987 | 1 | 0.987 |
|  | rs2070529 (T/C) | 0.869 | 1 | 0.892 | 1.029 (0.732-1.448) | 0.972 (0.691-1.367) | 0.063 | 0.693 | 0.116 |
|  | rs2070530 (C/G) | 0.892 | 1 | 0.892 | 1.024 (0.729-1.438) | 0.977 (0.695-1.372) | **0.003** | **0.033** | **0.015** |
|  | rs2070531 (C/T) | 0.239 | 1 | 0.527 | 0.806 (0.563-1.155) | 1.241(0.866-1.778) | 0.184 | 1 | 0.256 |
|  | rs6517481 (A/G) | 0.799 | 1 | 0.892 | 0.954 (0.662-1.373) | 1.049 (0.728-1.510) | 0.276 | 1 | 0.304 |
|  | rs7276961 (A/G) | 0.799 | 1 | 0.892 | 0.954 (0.662-1.373) | 1.049 (0.728-1.510) | 0.276 | 1 | 0.304 |
|  | rs1051475 (T/C) | 0.314 | 1 | 0.576 | 0.829 (0.576-1.194) | 1.206 (0.837-1.736) | **0.026** | **0.286** | **0.057** |
|  | rs1051476 (C/G) | 0.231 | 1 | 0.527 | 0.801 (0.557-1.152) | 1.249 (0.868-1.796) | **0.014** | **0.154** | **0.039** |
|  | rs11254 (C/T) | **0.04712** | 0.51832 | 0.259 | 1.487 (1.004-2.201) | 0.673 (0.454-0.996) | **0.0001** | **0.0011** | **0.001** |
|  | rs711 (G/A) | **0.00352** | **0.03872** | **0.039** | 1.858 (1.221-2.828) | 0.538 (0.354-0.819) | **0.004** | **0.044** | **0.015** |
| **ALL**  **(N=38)** | rs2269188 (G/C) | **0.01185** | 0.13035 | 0.065 | 2.17 (1.175-4.008) | 0.461 (0.250-0.851) | **0.041** | 0.451 | 0.226 |
|  | rs374575 (C/T) | 0.797 | 1 | 0.951 | 1.262 (0.416-3.826) | 0.792 (0.261-2.402) | 0.556 | 1 | 0.946 |
|  | rs2070529 (T/C) | 0.542 | 1 | 0.951 | 1.176 (0.698-1.981) | 0.85 (0.505-1.433) | 0.777 | 1 | 0.946 |
|  | rs2070530 (C/G) | 0.723 | 1 | 0.951 | 1.098 (0.655-1.839) | 0.911 (0.544-1.526) | 0.875 | 1 | 0.946 |
|  | rs2070531 (C/T) | 0.871 | 1 | 0.951 | 0.955 (0.551-1.657) | 1.047 (0.603-1.816) | 0.568 | 1 | 0.946 |
|  | rs6517481 (A/G) | 0.951 | 1 | 0.951 | 1.018 (0.583-1.775) | 0.983 (0.563-1.714) | 0.946 | 1 | 0.946 |
|  | rs7276961 (A/G) | 0.951 | 1 | 0.951 | 1.018 (0.583-1.775) | 0.983 (0.563-1.714) | 0.946 | 1 | 0.946 |
|  | rs1051475 (T/C) | 0.639 | 1 | 0.951 | 0.876 (0.504-1.522) | 1.141 (0.657-1.982) | 0.741 | 1 | 0.946 |
|  | rs1051476 (C/G) | 0.639 | 1 | 0.951 | 0.876 (0.504-1.522) | 1.141 (0.657-1.982) | 0.741 | 1 | 0.946 |
|  | rs11254 (C/T) | 0.543 | 1 | 0.951 | 0.844 (0.489-1.458) | 1.184 (0.686-2.045) | 0.394 | 1 | 0.946 |
|  | rs711 (G/A) | **0.00895** | 0.09845 | 0.065 | 2.617 (1.245-5.501) | 0.382 (0.182-0.803) | **0.02** | 0.22 | 0.220 |
| **BC**  **(N= 49)** | rs2269188 (G/C) | 0.131 | 1 | 0.616 | 1.478 (0.889-2.457) | 0.677 (0.407-1.125) | 0.31 | 1 | 0.757 |
|  | rs374575 (C/T) | 0.84 | 1 | 0.849 | 0.911 (0.371-2.238) | 1.097 (0.447-2.695) | 0.547 | 1 | 0.880 |
|  | rs2070529 (T/C) | 0.842 | 1 | 0.616 | 0.954 (0.599-1.518) | 1.048 (0.659-1.669) | 0.88 | 1 | 0.880 |
|  | rs2070530 (C/G) | 0.709 | 1 | 0.616 | 0.916 (0.577-1.454) | 1.092 (0.688-1.734) | 0.817 | 1 | 0.880 |
|  | rs2070531 (C/T) | 0.664 | 1 | 0.616 | 0.896 (0.546-1.470) | 1.116 (0.680-1.831) | 0.636 | 1 | 0.880 |
|  | rs6517481 (A/G) | 0.533 | 1 | 0.616 | 0.855 (0.523-1.399) | 1.169 (0.715-1.913) | 0.738 | 1 | 0.880 |
|  | rs7276961 (A/G) | 0.533 | 1 | 0.616 | 0.855 (0.523-1.399) | 1.169 (0.715-1.913) | 0.738 | 1 | 0.880 |
|  | rs1051475 (T/C) | 0.56 | 1 | 0.616 | 0.862 (0.523-1.421) | 1.16 (0.704-1.913) | 0.344 | 1 | 0.757 |
|  | rs1051476 (C/G) | 0.56 | 1 | 0.616 | 0.862 (0.523-1.421) | 1.16 (0.704-1.913) | 0.344 | 1 | 0.757 |
|  | rs11254 (C/T) | 0.384 | 1 | 0.616 | 0.804 (0.491-1.315) | 1.244 (0.760-2.036) | 0.178 | 1 | 0.757 |
|  | rs711 (G/A) | **0.00165** | **0.01815** | **0.018** | 0.469 (0.291-0.755) | 2.133 (1.324-3.437) | **0.002** | **0.022** | **0.022** |
| **OC**  **(N= 54)** | rs2269188 (G/C) | 0.132 | 1 | 0.559 | 1.454 (0.892-2.369) | 0.688 (0.422-1.121) | 0.333 | 1 | 0.686 |
|  | rs374575 (C/T) | 0.355 | 1 | 0.559 | 0.687 (0.309-1.529) | 1.455 (0.654-3.239) | 0.218 | 1 | 0.686 |
|  | rs2070529 (T/C) | 0.241 | 1 | 0.559 | 0.767 (0.492-1.196) | 1.304 (0.836-2.034) | 0.505 | 1 | 0.794 |
|  | rs2070530 (C/G) | 0.413 | 1 | 0.567 | 0.831 (0.533-1.295) | 1.204 (0.772-1.878) | 0.722 | 1 | 0.794 |
|  | rs2070531 (C/T) | 0.341 | 1 | 0.559 | 0.796 (0.497-1.274) | 1.257 (0.785-2.013) | 0.297 | 1 | 0.686 |
|  | rs6517481 (A/G) | 0.259 | 1 | 0.559 | 0.764 (0.478-1.221) | 1.309 (0.819-2.093) | 0.374 | 1 | 0.686 |
|  | rs7276961 (A/G) | 0.259 | 1 | 0.559 | 0.764 (0.478-1.221) | 1.309 (0.819-2.093) | 0.374 | 1 | 0.686 |
|  | rs1051475 (T/C) | 0.681 | 1 | 0.681 | 0.903 (0.556-1.468) | 1.107 (0.681-1.800) | 0.695 | 1 | 0.794 |
|  | rs1051476 (C/G) | 0.681 | 1 | 0.681 | 0.903 (0.556-1.468) | 1.107 (0.681-1.800) | 0.695 | 1 | 0.794 |
|  | rs11254 (C/T) | 0.621 | 1 | 0.681 | 0.886 (0.547-1.434) | 1.129 (0.698-1.828) | 0.862 | 1 | 0.862 |
|  | rs711 (G/A) | **0.04993** | 0.54923 | 0.549 | 1.758 (0.995-3.105) | 0.569 (0.322-1.005) | 0.091 | 1 | 0.686 |

C P = Crude P value, BF P = Bonferroni P value, BH P = Benjamini-Hochberg P value, OR = Odds Ratio, CI= 95% Confidence Interval

**Table S4. Comparative analysis of haplotypes in different study group**

| **Group** | **Haplotypes** | **Frequency in Test** | **Frequency in Control** | **Chi Sq (DF=1)** | **P value** | **Power (%)** |
| --- | --- | --- | --- | --- | --- | --- |
| **DSF** | A-C-C-C-T-C-C-A-A-T-C-C-C-G-G | 0.02619 | 0.06466 | 1.05 | 0.306 | 11.83 |
|  | A-C-C-C-T-C-C-A-A-T-C-G-A-G-A | 0.05079 | 1.91E-273 | 5.13 | **0.024** | 38.95 |
|  | A-C-T-C-C-G-T-G-G-C-G-C-C-G-G | 0 | 0.01423 | 1.01 | 0.316 | 11.56 |
|  | A-C-T-C-T-C-C-A-A-T-C-G-C-A-G | 0 | 0.01209 | 1.01 | 0.316 | 11.56 |
|  | G-C-C-C-T-C-C-A-A-C-G-C-A-G-A | 0.007717 | 0 | 1.01 | 0.316 | 11.56 |
|  | G-C-C-C-T-C-C-A-A-T-C-C-C-A-G | 0.01205 | 0 | 1.01 | 0.316 | 11.56 |
|  | G-C-T-C-C-G-T-G-G-C-G-C-C-A-G | 0.04347 | 8.51E-136 | 4.08 | **0.043** | 32.21 |
|  | G-C-T-C-C-G-T-G-G-C-G-G-C-G-G | 0 | 0.04605 | 5.13 | **0.024** | 38.95 |
|  | G-T-C-T-C-G-C-A-A-T-C-G-A-G-A | 0 | 0.01732 | 2.02 | 0.155 | 18.36 |
|  | G-T-T-C-C-G-T-G-G-C-G-G-A-A-G | 0.01726 | 0 | 2.02 | 0.155 | 18.36 |
| **DSM** | A-C-C-C-T-C-C-A-A-C-G-G-C-A-G | 0.01793 | 0 | 2.02 | 0.155 | 15.23 |
|  | A-C-C-C-T-C-C-A-A-T-C-C-A-A-G | 0 | 0.01216 | 1.01 | 0.316 | 10.03 |
|  | A-C-C-C-T-C-C-A-A-T-C-C-A-G-G | 0 | 0.01569 | 2.02 | 0.155 | 15.23 |
|  | A-C-C-C-T-C-C-A-A-T-C-C-C-A-G | 0 | 0.03849 | **4.08** | **0.043** | 26.00 |
|  | A-C-C-C-T-C-C-A-A-T-C-C-C-G-G | 0.04173 | 0.06466 | 0.421 | 0.516 | 7.067 |
|  | A-C-C-C-T-C-C-A-A-T-C-G-C-G-A | 0.07724 | 0.01703 | 3.79 | 0.052 | 24.49 |
|  | A-C-T-C-T-C-C-A-A-T-C-G-C-A-G | 0 | 0.01209 | 1.01 | 0.316 | 10.03 |
|  | G-C-C-C-C-G-T-G-G-C-G-G-C-G-G | 0 | 0.01122 | 1.01 | 0.316 | 10.03 |
|  | G-C-T-C-C-G-T-G-G-C-G-G-C-G-A | 9.61E-11 | 3.721e-318 | 0 | 1.000 | 5.00 |
|  | G-T-C-T-C-G-C-A-A-T-C-C-C-G-A | 0 | 0.01389 | 1.01 | 0.316 | 10.03 |
| **DSP** | A-C-C-C-T-C-C-A-A-T-C-C-A-G-G | 0.06745 | 0.01569 | 2.71 | 0.100 | 17.33 |
|  | A-C-C-C-T-C-C-A-A-T-C-C-C-G-A | 0 | 0.01836 | 2.02 | 0.155 | 14.12 |
|  | A-C-C-C-T-C-C-A-A-T-C-C-C-G-G | 0.006113 | 0.06466 | **3.7** | **0.054** | 21.97 |
|  | A-C-T-C-C-G-T-G-G-C-G-C-C-G-G | 0 | 0.01423 | 1.01 | 0.316 | 9.485 |
|  | A-C-T-C-T-C-C-A-A-T-C-C-C-A-G | 0.01334 | 0 | 1.01 | 0.316 | 9.485 |
|  | A-C-T-C-T-C-C-A-A-T-C-G-A-G-G | 0.05133 | 0 | **5.13** | **0.024** | 28.61 |
|  | G-C-C-C-C-G-T-G-G-C-G-C-C-G-G | 0.02711 | 0.0001918 | 3.05 | 0.081 | 18.93 |
|  | G-C-C-C-C-G-T-G-G-C-G-G-A-G-G | 0.02427 | 0.007116 | 0.338 | 0.561 | 6.479 |
|  | G-C-C-C-T-C-C-A-A-T-C-C-C-A-G | 0.01556 | 0 | 2.02 | 0.155 | 14.12 |
|  | G-C-C-C-T-G-T-G-G-C-G-C-C-G-G | 0.007937 | 0 | 1.01 | 0.316 | 9.485 |
|  | G-C-T-C-C-G-T-G-G-C-G-C-C-A-A | 0 | 0.009609 | 1.01 | 0.316 | 9.485 |
|  | G-C-T-C-C-G-T-G-G-C-G-G-A-G-A | 0 | 0.01136 | 1.01 | 0.316 | 9.485 |
|  | G-C-T-C-C-G-T-G-G-C-G-G-A-G-G | 0 | 0.02317 | 2.02 | 0.155 | 14.12 |
|  | G-C-T-C-C-G-T-G-G-C-G-G-C-A-G | 0 | 0.01977 | 2.02 | 0.155 | 14.12 |
|  | G-T-C-C-C-G-T-G-G-C-G-C-A-G-A | 0.02381 | 0 | 2.02 | 0.155 | 14.12 |
|  | G-T-T-C-C-G-T-G-G-C-G-G-C-A-G | 0 | 0.01736 | 2.02 | 0.155 | 14.12 |
|  | G-T-T-C-C-G-T-G-G-T-C-G-C-G-G | 0.0119 | 0 | 1.01 | 0.316 | 9.485 |
| **ALL** | A-C-C-C-T-C-C-A-A-T-C-G-A-G-G | 0.2017 | 0.02733 | 14.2 | **0.000** | 99.09 |
|  | A-C-T-C-C-G-T-G-G-C-G-G-A-G-G | 0.05802 | 0 | 6.19 | **0.013** | 81.43 |
|  | G-T-C-T-C-G-C-A-A-T-C-G-C-G-A | 0.02778 | 1.25E-234 | 3.05 | 0.081 | 51.73 |
| **BC** | A-C-C-C-T-C-C-A-A-T-C-C-C-G-G | 0 | 0.06466 | 6.19 | **0.013** | 71.00 |
|  | A-C-T-C-T-C-C-A-A-T-C-G-A-G-G | 0.06847 | 0 | 7.25 | **0.007** | 77.64 |
|  | A-T-T-C-C-G-C-A-A-T-C-G-C-G-A | 0.025 | 0 | 3.05 | 0.081 | 42.25 |
|  | G-C-C-C-C-G-T-G-G-C-G-G-A-G-A | 0.1245 | 0.003875 | 12.8 | **0.000** | 95.09 |
|  | G-C-C-C-C-G-T-G-G-C-G-G-C-A-G | 0.0375 | 0 | 4.08 | 0.043 | 53.21 |
|  | G-C-C-C-T-C-C-A-A-T-C-G-C-A-G | 0.025 | 0 | 3.05 | 0.081 | 42.25 |
|  | G-C-T-C-T-C-C-A-A-T-C-G-C-G-G | 0.04091 | 0 | 4.08 | 0.043 | 53.21 |
|  | G-T-C-C-C-G-C-A-A-T-C-C-C-G-A | 0 | 0.03219 | 3.05 | 0.081 | 42.25 |
|  | G-T-C-T-C-G-C-A-A-C-G-C-C-A-A | 0.025 | 0 | 3.05 | 0.081 | 42.25 |
| **OC** | A-T-C-C-C-G-T-G-G-T-C-C-A-G-G | 0.01699 | 0 | 2.02 | 0.155 | 27.72 |
|  | A-T-C-T-C-G-C-A-A-T-C-G-C-A-G | 0.03243 | 0 | 3.05 | 0.081 | 39.01 |
|  | G-C-C-C-T-C-C-A-A-T-C-G-A-G-G | 0.02214 | 0.003533 | 2.02 | 0.155 | 27.72 |
|  | G-C-C-C-T-C-C-A-A-T-C-G-C-A-G | 0.01271 | 0 | 1.01 | 0.316 | 16.21 |
|  | G-C-C-C-T-C-C-A-A-T-C-G-C-G-G | 0.03028 | 0 | 3.05 | 0.081 | 39.01 |
|  | G-C-T-C-C-G-T-G-G-C-G-G-C-A-G | 0.1132 | 0.01977 | 6.66 | **0.010** | 6.66 |
|  | G-T-C-C-C-G-C-A-A-T-C-C-C-G-A | 0 | 0.03219 | 3.05 | 0.081 | 39.01 |
|  | G-T-C-T-C-G-C-A-A-T-C-G-C-A-G | 0.004602 | 0 | 0 | 1 | 5 |
|  | G-T-C-T-C-G-C-A-A-T-C-G-C-G-G | 0.009259 | 0 | 0 | 1 | 5 |
|  | G-T-T-C-C-G-T-G-G-C-G-C-C-G-A | 0.02778 | 0 | 3.05 | 0.081 | 39.01 |

DSF: Father of probands with DS, DSM: Mother of probands with DS, DSP: DS proband, significant p values are marked in bold
